# Structural Features, Physicochemical Properties, and In Vitro Digestibility of the Starch-Lipid Complexes Formed between High Amylose Starch and Stearic Acid or Potassium Stearate

**DOI:** 10.3390/foods13060859

**Published:** 2024-03-12

**Authors:** Yuheng Zhai, Hao Zhang, Shangyuan Sang, Bin Ren, Yongjun Yuan, Jiali Xing, Xiaohu Luo

**Affiliations:** 1 Zhejiang-Malaysia Joint Research Laboratory for Agricultural Product Processing and Nutrition, College of Food Science and Engineering, Ningbo University, Ningbo 315832, China; yuheng.zhai@outlook.com (Y.Z.); sangshangyuan@nbu.edu.cn (S.S.); 2 National Engineering Research Center for Cereal Fermentation and Food Biomanufacturing, Jiangnan University, Wuxi 214122, China; leslie198907@foxmail.com; 3 College of Food Science and Engineering, Lingnan Normal University, Zhanjiang 524048, China; renbin@nbu.edu.cn; 4 Key Laboratory of Grain and Oil Processing and Food Safety of Sichuan Province, College of Food and Bioengineering, Xihua University, Chengdu 610039, China; yyja9791@sina.com; 5 Ningbo Academy of Product and Food Quality Inspection (Ningbo Fibre Inspection Institute), Ningbo 315048, China

**Keywords:** high amylose starch, lipid, starch-lipid complexes, in vitro digestibility

## Abstract

Starch-lipid complexes were prepared from high amylose starch (HAS) with stearic acid (SA) or potassium stearate (PS) at different molar concentrations. The complexes (HAS-PS) formed between HAS and PS showed polyelectrolyte characteristics with ζ-potential ranging from −22.2 to −32.8 mV, and the electrostatic repulsion between anionic charges restricted the starch chain reassociation and facilitated the formation of V-type crystalline structures upon cooling. The hydrophobic effects enabled recrystallization of the SA, and the HAS-SA complexes exhibited weaker V-type crystalline structures than the HAS-PS complexes; both HAS-SA/PS complexes were of a similar “mass fractal” type, with a dimension varied from 2.15 to 2.96. The HAS-SA complexes had a considerable content of resistant starch (RS, 16.1~29.2%), whereas negligible RS was found in the HAS-PS complexes. The findings from the present study imply that the molecular order of starch chains and the macro-structures of starch particles are more important to regulate the digestibility of starch-lipid complexes than the crystalline structures.

## 1. Introduction

Starch is one of the most important energy sources for the human body, existing in the form of semi-crystalline granules composed of amylose and amylopectin. Amylose is a non-branched linear molecule connected by glucose molecules through α-1,4-glycosidic bonds, while amylopectin is a kind of starch polymer with a highly branched structure. When starch is heated in excess water, its granular structure is irreversibly destroyed, thereby releasing amylose, which can interact with a variety of ligands, including lipids [1], flavor compounds [2], and aroma molecules [3]. The amylose-ligand interactions generally generate inclusion complexes characteristic of a left-handed single-helical structure [4]. In recent years, studies have found that a complex of starch and lipid can effectively modify the physicochemical properties of starch, such as reducing the swelling power and solubility of starch, increasing gelatinization temperature, delaying retrogradation, and hindering the hydrolysis of enzymes [5,6]. Therefore, amylose-lipid complexes have been studied extensively in the food industry [7,8,9]. 

From a nutritional point of view, the digestibility of starch can be classified as rapidly digestible starch (RDS), slowly digestible starch (SDS), and resistant starch (RS) [10]. RS is the fraction that cannot be digested in the small intestine but can be fermented in the large intestine [11]. Studies have found that regular intake of RS can prevent chronic diseases such as obesity and promote intestinal health [11,12]. In the past decades, starch-lipid complexes have been recognized as a novel RS, referred to as RS5 [13,14]. Thus, the preparation of starch-lipid complexes has recently attracted great attention from scholars. Concerning the complex formation, many factors have been shown to influence the interactions between starches and lipids, such as temperature, pH, and reaction time [15,16,17]. Additionally, the structural characteristics of lipids and starches also contribute to the complexation [18]. In a previous study, Chao et al. [19] investigated the complexes of starch with fatty acids and their mono-, di-, and triglycerides. The results suggested that the complexing index followed the order of monoglyceride > fatty acids > triglyceride > diglyceride, and the difference in the complexing index was attributed to the steric hindrance effects and water solubility of lipids. Moreover, Niu et al. [20] reported that adding NaCl could promote the formation of complexes between starch and fatty acids due to the salt-induced dissolution of fatty acids. Fatty acids can exist in the form of fatty acid salts, which have improved water-solubility over fatty acids. To the best of our knowledge, few studies have been devoted to how different forms of fatty acids affect the formation of starch-lipid complexes. The purpose of the present work was to investigate the complexation between starch and fatty acids or fatty acid salts, and to evaluate the changes in the physicochemical and nutritional properties of these complexes. The results could be of great significance in the preparation of starch-lipid complexes of different physicochemical properties toward various applications in the food industry.

## 2. Materials and Methods

### 2.1. Materials

High amylose starch (HAS, AmylogelTM 03003) was kindly donated by Cargill Investments Ltd. (Shanghai, China). The content of amylose in the HAS was determined to be ca. 75% using a colorimetric method [21]. Isoamylase (Cas: 9067-73-6), pancreatic α-amylase (Cas: 9000-90-2), amyloglucosidase (Cas: 9032-08-0) from Aspergillus niger, and glucose oxidase/peroxidase reagent (GOPOD) were purchased from Megazyme (Wicklow, Ireland) and used as received. Stearic acid (SA) and potassium stearate (PS) were of analytical grade (Sinopharm Chemical Reagent Co., Ltd., Shanghai, China).

### 2.2. Preparation of Starch-Lipid Dispersions

The starch-lipid dispersions were prepared using a high-temperature reactor (MC500, Beijing Century Senlong experimental apparatus Co., Ltd., Beijing, China). Briefly, 4.0 g of HAS was mixed with PS or SA at different molar concentrations (0.5 mM, 1.0 mM, 1.5 mM, and 2.0 mM) in 100 mL deionized water. The suspensions were heated to 140 °C at a rate of *ca.* 10 °C/min and held at 140 °C for 20 min with constant stirring (150 rpm). When the dispersions were cooled to 80 °C, they were transferred to a beaker and incubated in a water bath at 25 °C for 2 h before further analysis. To simplify the description, the dispersions were noted as HAS-SA/PS-K (m), where K represents the molar concentrations of SA/PS and m represents the mass ratio of HAS to SA/PS, respectively. The blank control (without the addition of SA/PS) was prepared using the same protocol.

### 2.3. Zeta (ζ)-Potential of the Dispersions

A Zetasizer (ZEN3600, Malvern Instruments, Ltd., Worcestershire, UK) was used to determine the zeta potential of the HAS-SA/PS dispersions. Before measurement, the dispersions were diluted to 0.1 mg/mL.

### 2.4. Rheological Properties

Dynamic rheological properties of the HAS-SA/PS mixtures were investigated using a DHR-3 rheometer (TA Instruments, New Castle, DE, USA). A 40 mm parallel plate geometry with a gap of 1000 μm was used for the test. [22]. Before the measurements, the HAS-SA/PS dispersions were stored in a 4 °C refrigerator for 48 h. The samples were transferred to the testing plate and equilibrated at 25 °C for 30 s. To prevent water evaporation of the samples, a layer of mineral oil was coated around the rim. To reveal the linear viscoelastic regime, a strain sweep test was performed within the range of 0.01~100%, and the frequency for measurements was selected as 1 Hz. In the case of frequency sweep tests, the HAS-SA/PS dispersions were scanned with the frequency ranging from 1 rad/s to 100 rad/s at an oscillation strain amplitude of 0.1%, which was in the linear viscoelastic regime. All experiments were carried out at 25 °C.

### 2.5. Isolation of Starch-Lipid Complexes

After storing in the refrigerator, the HAS-SA/PS dispersions were centrifuged at 6000× *g* for 15 min to separate the starch-lipid complex. The isolated pellets were washed 3 times using deionized water to remove residual SA or PS. Then, the samples were immediately freeze-dried and milled to pass through a 100-mesh sieve (0.15 mm).

### 2.6. X-ray Diffraction (XRD) Analysis

Before the experiments, the freeze-dried samples were stored in a hermetic chamber with 100% relative humidity for 24 h to balance moisture. Then, X-ray diffraction analysis of samples was performed using a D8 diffractometer (Bruker, Krlsruhe, Germany) operating at 45 kV and 30 mA with Cu Ka radiation (λ = 1.54 Å) according to the method of a previous study [23] with some modifications. The samples were scanned from 5° to 40° at a scan rate of 4°/min. The data were analyzed using Jade 6.0 software (Materials Data Inc., Livermore, CA, USA), and the relative crystallinity (*R_c_*) was calculated as follows:

(1) $$R_{c}(\%)=\frac{A_{c}}{A_{c}+A_{a}}\times 100$$

where *A_c_* represents the crystalline area of the XRD patterns and *A_a_* represents the amorphous area of the XRD patterns, respectively.

### 2.7. Fourier Transform Infrared (FTIR) Spectroscopy

The FTIR spectra of freeze-dried powders of starch-lipid complexes were observed using a spectrometer (Thermal Fisher Nicolet Co. Ltd., Waltham, MA, USA) equipped with an ATR accessory. The spectra were collected from 4000 cm^−1^ to 400 cm^−1^ by accumulating 64 scans at a resolution of 4 cm^−1^ against air as the background. The infrared spectra were analyzed using OMNIC 8.0 software (Thermo Fisher Scientific, Inc., Waltham, MA, USA). A half bandwidth of 19 cm^−1^ and a resolution enhancement factor of 1.9 were used [24].

### 2.8. Small Angle X-ray Scattering (SAXS)

The small angle X-ray scattering (SAXS) measurement was carried out using an SAXS point 2.0 system (Anton Paar, Graz, Austria) with a Microsource X-ray source, producing Cu Kα radiation at a wavelength (λ) of 0.154 nm. Prior to the measurements, the freeze-dried powders were hydrated with deionized water at a moisture content of ca. 55% (*w*/*w*) and equilibrated at 4 °C for 24 h. Then, the hydrated samples were placed on a sample holder and sealed hermetically. The data were collected in the range of 0.03 < *q* < 4.5 nm^−1^, where *q* = 4π sin2θ/λ (2θ represents the scattering angle).

### 2.9. Scanning Electron Microscopy (SEM)

The micrographs of starch-lipid complexes were observed using a field emission scanning electron microscope (S-4800, Hitachi Science Systems, Ltd., Tokyo, Japan) under an accelerating voltage of 5 kV. Before observation, the samples were sprinkled on a double-sided adhesive tape, followed by coating with a thin layer of gold (ca. 10 nm) under vacuum.

### 2.10. Confocal Laser Scanning Microscopy (CLSM)

A confocal laser scanning microscope (LSM880, Carl Zeiss Inc., Braunschweig, Germany) was used to observe the distribution of lipids in the starch-lipid complexes. In brief, 20 mg of freeze-dried starch-lipid complexes were weighed and suspended in 1.0 mL of Nile red solution (0.1%, *w*/*v* in ethanol). The sample was kept at 4 °C for 12 h, followed by centrifugation at 10,000× *g* for 5 min to remove the supernatant. Then, the Nile red-stained mixtures were placed on a glass slide and observed using the microscope.

### 2.11. In Vitro Digestibility

Before digestion, the content of starch in the complexes was first determined. In brief, 100 mg of starch-lipid complexes (dry basis) were suspended in 2 mL of 2.0 M potassium hydroxide, and the mixtures were stirred for 30 min in an ice-water bath. Then, 8.0 mL of 1.2 M sodium acetate buffer (pH 3.8) was added to the mixtures and stirred vigorously. Subsequently, 0.2 mL of amyloglucosidase (3300 U/mL) was added to the test tube and incubated in a 50 °C water bath for 12 h. The total glucose in the mixtures was determined using the GOPOD kit, and the content of starch was calculated as follows:

(2) $$Starch\;content\left(\%\right)=\frac{m_{glu}\times 0.9}{m_{ini}}\times 100$$

where *m_glu_* represents the total weight of released glucose, *m_ini_* represents the initial weight of the starch-lipid complexes, and 0.9 represents the transformation coefficient from glucose to starch in weight.

The in vitro digestibility of starch-lipid complexes was determined according to the Englyst method with some modifications [25]. Briefly, the samples (containing 100 mg starch, dry basis) were weighed and added to a screw-cap tube. Subsequently, 5 mL of the digestive juice was prepared by dissolving α-amylase and amyloglucosidase into a 0.1 M sodium acetate buffer (with 4 mM CaCl_2_, pH 5.2) at final concentrations of 290 U/mL and 15 U/mL, respectively. Before being added, the juice was held in a 37 °C water bath for 10 min to activate the enzymes. Then, the mixture was shaken at a speed of 160 r/min in a 37 °C water bath. At the designated time, a 50 μL aliquot of the sample solution was taken into a 2 mL microcentrifuge tube and then 450 μL of absolute ethanol was added to deactivate the digestive enzymes, followed by centrifugation at 10,621× *g* for 2 min. The released glucose content was measured using the GOPOD kit, and the digested starch content was calculated using the following formula:

(3) $$Digested\;\mathrm{starch}\left(\%\right)=\frac{m_{glu}\times 0.9}{m_{s}}\times 100$$

where *m_glu_* represents the total weight of released glucose in the supernatant, *m_s_* represents the initial weight of added starch, and 0.9 represents the transformation coefficient from glucose to starch in weight.

### 2.12. Statistical Analysis

Each test was performed independently, in triplicate, unless otherwise specified, and the data were reported as mean ± standard deviation. Statistical significance was analyzed with a one-way ANOVA followed by Duncan’s test at *p* < 0.05 (SPSS 21.0, SPSS Inc., Chicago, IL, USA).

## 3. Results

### 3.1. Formation of HAS-SA/PS Dispersions and Rheological Properties

**Figure 1:**
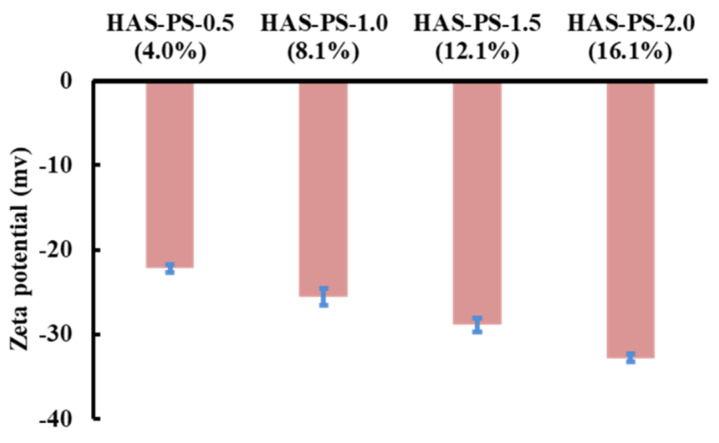
Zeta-potential of HAS-PS dispersions.

To facilitate the formation of starch-lipid complexes, the HAS was gelatinized in a high-temperature reactor to release the amylose molecules. After thermal treatment, the control, as well as the HAS-SA/PS, showed viscous dispersions without visible particles, and the addition of PS improved the clarity of the HAS-PS dispersions. Results from the ζ potential showed that the HAS-PS dispersions were negatively charged (Figure 1), while the HAS-SA dispersions did not show polyelectrolyte characteristics. Additionally, the magnitude of the negative ζ potential of HAS-PS dispersions gradually increased from −22.2 mV to −32.8 mV when the addition of PS increased from 0.5 mM to 2.0 mM (Figure 1). In a previous study [22], the starch-lipid complexes prepared from chain-elongated amylopectin and sodium palmitate also showed polyelectrolyte characteristics, and structural analysis revealed that the hydrophobic hydrocarbon chain of sodium palmitate is trapped in the starch particles via a V-type helix formation, and the carboxylate group was present on the particle surface. Consequently, a higher magnitude of ζ potential might result from a greater inclusion of PS within the HAS-PS particles.

**Figure 2:**
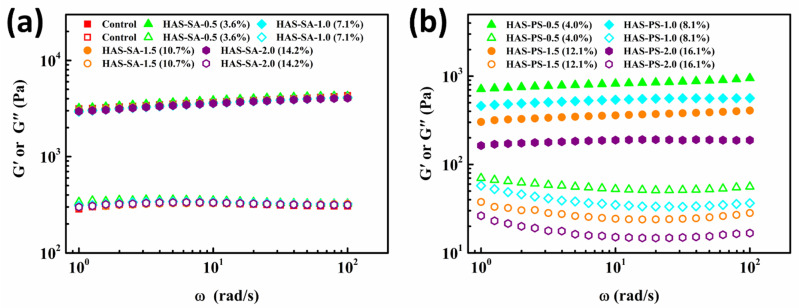
Mechanical spectra of dispersions containing 4.0% (*w*/*v*) high amylose starch (HAS) with (**a**) stearic acid (SA) or (**b**) potassium stearate (PS) at molar concentrations of 0.5~2.0 mM. The solid symbols represent the *G*′, and the open symbols indicate the *G*″. Before rheological tests, all of the dispersions were stored at 4 °C for 48 h.

Upon cooling in the refrigerator, all HAS-SA/PS dispersions, including the control, developed gels. In general, the structural properties of gels can be characterized by dynamic rheological testing. The storage modulus (*G*′) measures the energy stored as mechanical energy, whereas the loss modulus (*G*″) indicates the energy dissipated as heat, mostly representing the viscous portion [26]. For the control, the *G*′ was almost ten times higher than the *G*″, and the value of *G*′ was nearly independent of the frequency at the test range (Figure 2a), indicating an elastic-dominant behavior [27]. Relative to the control, the addition of SA did not change the rheological properties of HAS-SAs significantly (Figure 2a). Moreover, the magnitudes of *G*′ and *G*″ were independent of the SA proportion, suggesting that the addition of SA did not significantly change the gel structures of HAS-SAs, which might result from a poor inclusion capability of starch with SA.

On the contrary, the addition of PS remarkably altered the rheological properties of HAS-PS (Figure 2b), although the HAS-PSs had similar elastic-dominant behavior with respect to the control sample. The gel strength of HAS-PSs was highly dependent on the added PS. With increasing PS, the magnitudes of *G*′ and *G*″ of HAS-PSs gradually decreased (Figure 2b). The formation of starch gels involves the reassociation of starch chains, where the hydrogen bonding formed between adjacent starch chains determines the gel strength. According to previous studies [22,28], the addition of sodium palmitate decreased the viscosity of the amylose solution, and no visual or microscopic evidence of retrogradation was observed for amylose because the electrostatic repulsion restricted the amylose reassociation. In this study, the hydrophobic forces transferred the hydrophobic chain of PS into the amylose helix cavity, and the ionization of −COOK provided the polyelectrolyte characteristics of HAS-PS (Figure 1). The electrostatic repulsion between anionic charges was the main reason that the reassociation of starch chains decreased during storage in the refrigerator, which was reported in a previous study [22], and thus a higher content of PS would lead to a weaker gel strength of HAS-PS.

### 3.2. Crystalline Structures Resolved by XRD

**Figure 3:**
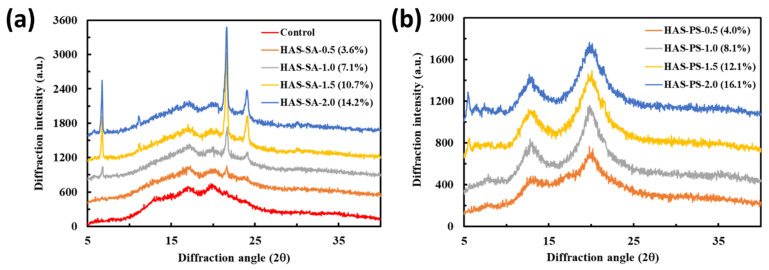
X-ray diffraction patterns of freeze-dried powders from dispersions containing 4.0% (*w*/*v*) high amylose starch (HAS) with (**a**) stearic acid (SA) or (**b**) potassium stearate (PS) at a molar concentration of 0.5~2.0 mM. The control was prepared from the dispersion containing 4.0% (*w*/*v*) HAS, but without the addition of SA or PS.

To further explore the interactions between HAS and SA/PS, XRD patterns of freeze-dried HAS-SA/PS were characterized. In the present study, the XRD pattern of the control showed major diffraction peaks at 2θ of 17.1° and 19.9° (Figure 3a), revealing a typical B+V crystalline structure [22]. The formation of a V-type crystalline structure was related to the presence of lipids in native HAS (the content of lipids was *ca.* 0.7%). Freeze-dried HAS-SA-0.5 (3.6%) had three obvious diffraction peaks at 2θ of 17.1°, 19.9°, and 21.6°, and a small one at 2θ of 23.9° (Figure 3a). The diffraction peak at 2θ of 17.1° is indicative of a B-type crystalline structure [29], whereas the diffraction peak at 2θ of 19.9° is characteristic of V-type crystalline polymorphs formed between amylose and SA [22]. Figure S1 shows the XRD patterns of SA and PS, and two sharp diffraction peaks at 2θ of 21.6° and 23.9° were observed for SA. Thus, the diffraction peaks in HAS-SA at 2θ of 21.6° and 23.9° indicated the recrystallization of free SA. In the case of HAS-SA-1.0 (7.1%), HAS-SA-1.5 (10.7%), and HAS-SA-2.0 (14.2%), two additional diffraction peaks at 2θ of 6.7° and 11.2° appeared (Figure 3a), resulting from the recrystallized SA (Figure S1). With the increasing addition of SA from 0.5 mM to 2.0 mM, the *R_c_* of HAS-SAs increased from 22.7% to 33.2% (Table 1). Nevertheless, it should be noted that increased SA did not change the peak intensity at 2θ of 17.1° and 19.9°, but gradually increased the peak intensity at 2θ of 6.7°, 21.6°, and 23.9° (Figure 3a), suggesting that the B- and V-type crystallinity of HAS-SAs was independent of the amount of SA added. In a previous study, Li et al. [30] investigated the effects of fatty acid type (myristic, palmitic, stearic, oleic, linoleic, and linolenic acid) on the properties of starch-lipid complexes. The results implied that complexes prepared with SA had the lowest V-type crystallinity.

For HAS-PS-0.5 (4.0%), two major diffraction peaks at 2θ of 12.8° and 19.9° were observed (Figure 3b), which could attribute to the formation of V-type crystalline structures [22]. In addition, a small and unresolved peak at 2θ of 17.1° was identified (Figure 3b), revealing negligible B-type crystallites. When the PS increased to 1.0 mM, the diffraction peak at 2θ of 17.03° disappeared, and only two major peaks at 2θ of 12.82° and 19.95° were observed for HAS-PS-1.0 (8.1%) (Figure 3b). Additionally, the peak intensities at 2θ of 12.82° and 19.95° were remarkably improved, suggesting that the addition of PS impeded the reassociation of starch chains and facilitated the development of V-type crystalline structures. Further increased PS did not significantly alter the peak intensities at 2θ of 12.82° and 19.95° for HAS-PS-1.5 (12.1%) or HAS-PS-2.0 (16.1%) (Figure 3b). Additionally, extra-small diffraction peaks at 2θ of 5.6°, 6.4°, and 7.5° were observed, ascribed to the slight recrystallization of uncomplexed PS (Figure S1). These observations indicated that the addition of 1.5 mM or 2.0 mM PS might be saturated for the development of V-type crystalline structures under the studied conditions. When the same amount of SA or PS was added, PS was more likely to form a V-shaped crystalline structure with amylose than SA (Figure 3). This may be due to the fact that the electrostatic repulsion restricted the aggregation of PS upon cooling, whereas SA would be apt to aggregate by hydrophobic attractions [31].

### 3.3. FTIR Analysis

**Figure 4:**
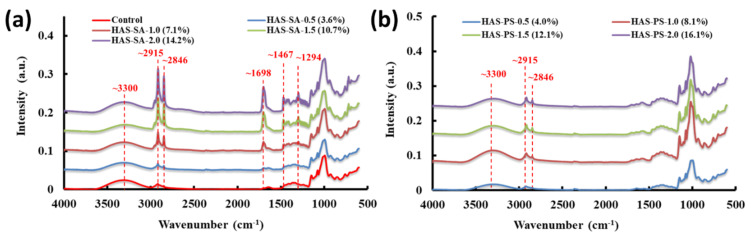
FTIR spectra of freeze-dried powders from dispersions containing 4.0% (*w*/*v*) high amylose starch (HAS) with (**a**) stearic acid (SA) or (**b**) potassium stearate (PS) at molar concentrations of 0.5~2.0 mM. The control sample was prepared from the dispersion containing 4.0% (*w*/*v*) HAS, but without the addition of SA or PS.

The FTIR spectra of freeze-dried HAS-SAs and HAS-PSs are shown in Figure 4. All spectra exhibited a broad band at approximately 3300 cm^−1^, owing to the symmetrical stretching vibration of O−H and C−H groups [32]. For the spectra of HAS-SAs (Figure 4a), a sharp band at ~2915 cm^−1^ was accompanied by a small band appearing at ~2846 cm^−1^, resulting from the asymmetrical and symmetrical stretching vibrations of the −CH_2_− group, respectively [33]. The band at 1698 cm^−1^ was assigned to the stretching vibration of the C=O unit of the carbonyl group, while the band at 1467 cm^−1^ was associated with the asymmetrical bending of the −CH_3_ group [33]. All the bands are characteristic of fatty acids, and the intensities of these bands gradually enhanced with the increased addition of SA from 0.5 mM to 2.0 mM. Figure S2a shows the enlarged spectra of HAS-SAs at 1200~800 cm^−1^. In general, the bands at ~995 cm^−1^ and ~1047 cm^−1^ correspond to the molecular order and crystallinity of starch, while the band at ~1022 cm^−1^ is related to the disorder or amorphous phase [34]. The absorbance ratios at both (1047/1022) cm^−1^ and (995/1022) cm^−1^ can be used to characterize the degree of order in starches [24,34]. As shown in Figure S2a, the band at ~1022 cm^−1^ shifted to a higher wavenumber of ~1016 cm^−1^, presumably due to the structuring effect of water on starch polymers. After deconvolution, the absorbance ratios at (1047/1016) cm^−1^ and (995/1016) cm^−1^ were calculated and listed in Table 1. All HAS-SAs showed similar absorbance ratios at (1047/1016) cm^−1^ and (995/1016) cm^−1^, suggesting that the molecular order of starch chains is scarcely affected by the added SA.

Figure 4b shows the spectra of freeze-dried HAS-PSs. With the PS increased from 0.5 to 2.0 mM, the band intensities at 2915 cm^−1^ and 2816 cm^−1^ were slightly increased. In addition, the HAS-PSs had much lower band intensities compared to the corresponding HAS-SAs. Furthermore, the characteristic bands for fatty acids at 1698 cm^−1^, 1467 cm^−1^, and 1294 cm^−1^ were not detected for HAS-PSs. The XRD results implied the extensive formation of V-type crystalline structures in HAS-PSs. Comparing the FTIR spectra between HAS-SAs and HAS-PSs, it can be inferred that the improved band intensity of fatty acids in HAS-SA spectra could be attributed to the uncomplexed SAs, and the development of V-type structures would eliminate the chemical bond vibration of PS. Figure S2b shows the enlarged spectra of HAS-PSs, and a similar band shifting at 1019 cm^−1^ was observed for HAS-PSs. Increasing the addition of PS seemed to enhance the band intensity at 1019 cm^−1^. HAS-PS-0.5 (4.0%) showed higher absorbance ratios at (1047/1022) cm^−1^ and (995/1022) cm^−1^ than HAS-PS-1.0 (8.1%), suggesting a greater molecular order of starch polymer in HAS-PS-0.5 (4.0%), although it showed lower *R_c_*. In addition, no significant difference in absorbance ratios was observed in HAS-PS-1.0 (8.1%), HAS-PS-1.5 (12.1%), or HAS-PS-2.0 (16.1%), and the HAS-SAs showed a higher absorbance ratio at (995/1022) cm^−1^ than the corresponding HAS-PSs. Therefore, these results imply that band absorbance at the range of 1200~800 cm^−1^ mainly reflected the molecular order of starch polymers (such as the alignment of double helices) rather than starch-lipid complexes.

### 3.4. SAXS Analysis

**Figure 5:**
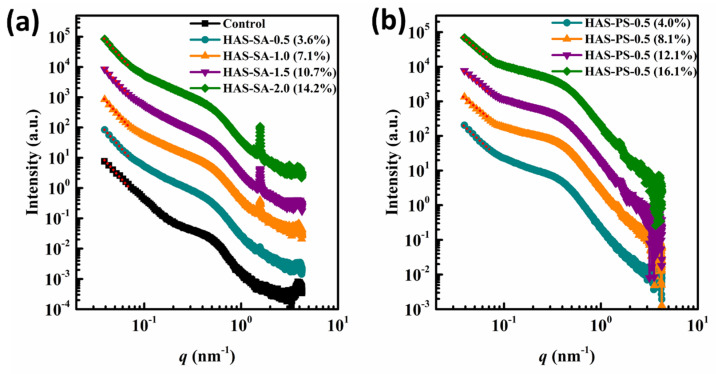
SAXS profiles of hydrated control and HAS-SAs (**a**) and HAS-PSs (**b**). The HAS-SAs/PSs were prepared from dispersions containing 4.0% (*w*/*v*) high amylose starch (HAS) with stearic acid (SA) or potassium stearate (PS) at molar concentrations of 0.5~2.0 mM. The control sample was prepared from a dispersion containing 4.0% (*w*/*v*) HAS, but without the addition of SA or PS.

In this study, thermal treatment destroyed the crystalline structure of native starch granules. Although it is hard to assign the peak positions unequivocally, the control showed a shoulder peak at q of *ca.* 0.50 nm^−1^ (Figure 5a), suggesting that the retrogradation of starch molecules also formed a lamellar structure with a periodic length of ca. 12.6 nm according to the Bragg’s law (d-spacing, d = 2π/q). Moreover, a small peak at q of ca. 4.0 nm^−1^ was identified, corresponding to a 100 inter-helix reflection of B-type crystalline structures [35], which agreed well with the results of XRD. For HAS-SAs, the addition of SA did not significantly change the peak position and intensities at q of ca. 0.50 nm^−1^ and ca. 4.0 nm^−1^ (Figure 5a). Nevertheless, an additional scattering peak at q of 1.56 nm^−1^ was observed for all HAS-SAs, which gradually enhanced when the addition of SA increased from 0.5 mM to 2.0 mM (Figure 5a), which could be attributed to the uncomplexed SA [36]. In the case of HAS-PSs, all SAXS profiles showed a shoulder peak at q of ca. 0.4 nm^−1^ (Figure 5b), corresponding to a periodic length of ca. 15.7 nm. Zabar et al. [37] reported similar shoulder peaks for amylose-SA complexes, and they suggested that the alternation of V-type crystalline and amorphous layers led to the lamellar structures in the amylose-SA complexes, and the peak position reflected the thickness of crystalline and amorphous layers. On the other hand, the difference in PS content did not significantly alter the peak position and intensity at q of 0.4 nm^−1^, whereas the scattering peak at q of 4.0 nm^−1^ disappeared for HAS-PS-1.0 (8.1%), HAS-PS-1.5 (12.1%), and HAS-PS-2.0 (16.1%) (Figure 5b), suggesting that the addition of PS inhibited the formation B-type crystalline structures. In addition, HAS-PS-1.5 (12.1%) and HAS-PS-2.0 (16.1%) showed a small scattering peak at q of 1.52 nm^−1^ (Figure 5b), resulting from the slight recrystallization of PS, as suggested by XRD. When compared to corresponding HAS-SAs, the shoulder peak intensity of HAS-PSs became greater, meaning a more ordered lamellar structure.

In general, the scattering patterns from a fractal object can be described by the power law *I(q)*∝*q^α^*, where *I(q)* is the scattering intensity and *q* is the scattering vector [38,39]. In the case of −3 < *α* < −1, the scattering source is assigned as a ‘mass fractal’, which means that the density profile of scattering objects is self-similar and the mass fractal dimension (*D_m_*) is obtained as follows: *D_m_* = −*α* [39]. The aggregation of polymers or particles with fractal characteristics creates a non-integer *D_m_* representing the degree of compactness [39]. As shown in Figure 5, all HAS-SAs/PSs showed a good linearity between *q* and *I*(*q*) at low-*q* regions (*ca.* 0.039 nm^−1^ ≤ *q* ≤ *ca.* 0.1030 nm^−1^) on the log-log graph, which was similar to results reported previously [37]. Also, the value of α varied from −2.96 to −2.15, revealing that the physical arrangement of starch polymers is similar to a “mass fractal” at the studied conditions. From Table 1, it can be seen that the *D_m_* of HAS-SAs was independent of the amount of SA added, suggesting that SA was not involved in the chain rearrangement of starch. Therefore, the HAS-SAs of different SA additions had a similar degree of compactness. Nevertheless, when the addition of PS increased from 0.5 mM to 2.0 mM, the *D_m_* of HAS-PSs decreased from 2.56 (HAS-PS-0.5 (4.0%)) to 2.15 (HAS-PS-2.0 (16.1%)), implying that the addition of PS restricted the chain reassociation of starch. Thus, a decrease in compactness was observed for HAS-PSs. On the other hand, the *D_m_* of HAS-PSs was lower than that of corresponding HAS-SAs, revealing fewer compact structures of HAS-PSs. Overall, these results suggest that the anionic charges of PS, including in amylose, created an electrostatic repulsion between neighboring starch chains, whereby the hydrogen bonding was weakened.

### 3.5. Morphology of Freeze-Dried Starch-Lipid Complexes

**Figure 6:**
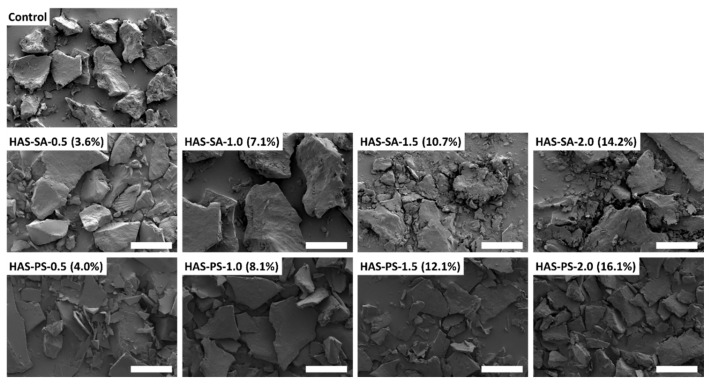
SEM images of freeze-dried powders from dispersions containing 4.0% (*w*/*v*) high amylose starch (HAS) with stearic acid (SA) or potassium stearate (PS) at molar concentrations of 0.5~2.0 mM. Scaling bar = 100 μm.

Figure 6 shows that the freeze-dried HAS-SAs present a typical appearance of retrograded starch, with an irregular shape with sharp edges [40]. The dimensions of the particles were mostly larger than 50 μm after grinding. Increasing the content of SA in HAS-SAs did not change the size, shape, or appearance of these particles. On the other hand, the morphology of freeze-dried HAS-PSs differed from the control or corresponding HAS-SAs, and a stack of thin sheets was observed for all HAS-PSs (Figure 6). The thickness for some of the sheets was around 2~5 μm, and the difference in PS content had limited effects on the morphology of HAS-PSs. From Figure 6, it can be seen that the HAS-PSs had less compact structures than the corresponding HAS-SAs. As suggested by rheological and SAXS analysis, this was due to the lesser extent of starch chain reassociation in HAS-PSs.

**Figure 7:**
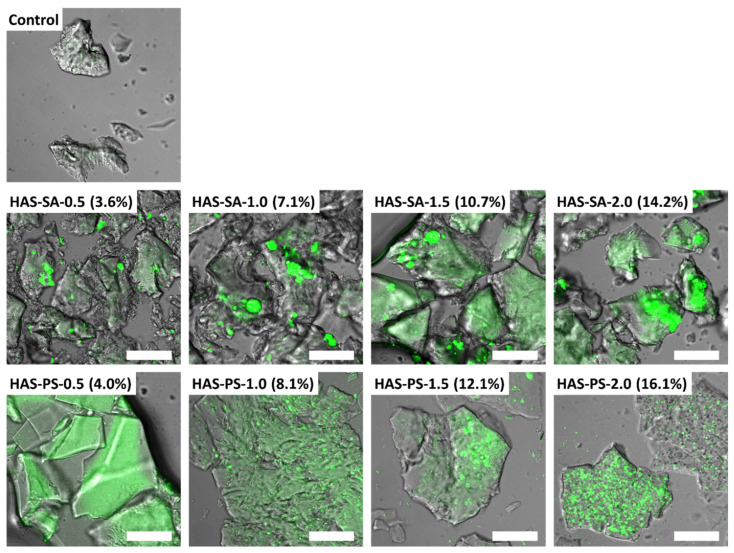
CLSM images of the Nile red-stained control and HAS-SAs/PSs. The HAS-SAs/PSs were prepared from dispersions containing 4.0% (*w*/*v*) high amylose starch (HAS) with stearic acid (SA) or potassium stearate (PS) at molar concentrations of 0.5~2.0 mM. The control was prepared from a dispersion containing 4.0% (*w*/*v*) HAS, but without the addition of SA or PS. Scaling bar = 100 μm.

The distribution of SA or PS in HAS-SAs/PSs was characterized by CLSM. Figure 7 shows the images of Nile red-stained HAS-SAs and HAS-PSs. In all the images, the SA and PS were dyed using green fluorescence. The fluorescence intensity became strengthened in the HAS-SAs particles with the increased addition of SA. Certain areas within the sample matrix were overexposed due to the regional assembly of uncomplexed SA, in accordance with the findings of Marinopoulou et al. [15], who speculated that the overexposure of certain areas in amylose-fatty acid complexes could be attributed to free fatty acid crystals entrapped between the lamellae of the complexed amylose. In the case of HAS-PSs, however, the PS was fairly well distributed throughout the matrix of the particles, resulting from the inclusion of PS in the amylose helix. Some strong fluorescent spots were also observed in HAS-PS-1.5 (12.1%) and HAS-PS-2.0 (16.1%) because of the recrystallization of excessive PS, as indicated by XRD. Compared to the SA distributed in HAS, the distribution of PS in the HAS matrix at the same quantity showed a more rigorous ordering, presumably due to the fact that the anionic charges of free PS prevented the excessive aggregation of PS in the sample matrix.

### 3.6. In Vitro Digestion

**Figure 8:**
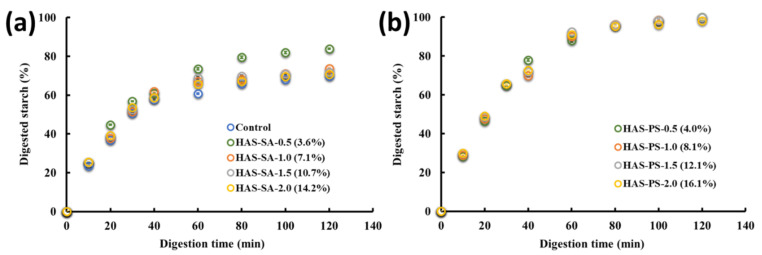
In vitro digestion profiles of control and HAS-SAs (**a**), and HAS-PSs (**b**). The HAS-SAs/PSs were prepared from dispersions containing 4.0% (*w*/*v*) high amylose starch (HAS) with stearic acid (SA) or potassium stearate (PS) at molar concentrations of 0.5~2.0 mM. The control was prepared from a dispersion containing 4.0% (*w*/*v*) HAS, but without the addition of SA or PS.

The in vitro digestion profiles of the control, HAS-SAs, and HAS-PSs are illustrated in Figure 8. For the control and HAS-SAs, a fast increase of the digested starch was observed at the initial 30 min, and the digestion rate became slower at the following stage (40~120 min). When digestion reached 120 min, the percentage of digested starch for HAS-SA-0.5 (3.6%), HAS-SA-1.0 (7.1%), HAS-SA-1.5 (10.7%), and HAS-SA-2.0 (14.2%) was determined to be 83.9%, 73.7%, 71.9%, and 70.8%, respectively (Figure 8a). According to the Englyst method, the digested fraction in the initial 20 min is RDS, while SDS refers to the digested fraction between 20 min and 120 min, and the RS represents the undigested remnants after 120 min [10]. As shown in Table 1, the RDS, SDS, and RS contents in the control were 36.7%, 33.0%, and 30.3%, respectively. Compared to the control, the addition of SA increased the content of RDS but decreased the content of RS in the HAS-SAs (Table 1), irrespective of the amount of SA added. On the other hand, increasing the addition of SA gradually increased the content of RS (Table 1). Structural characterizations revealed that the addition of SA did not significantly change the B- or V-type crystallinity, molecular order, or lamellar stacking of HAS-SAs. However, CLSM images show the aggregation of SA in the HAS-SA matrix. Consequently, it is possible that the decreased digestibility of HAS-SAs might be attributed to the physical encapsulation of starch polymers via SA, which would lower the accessibility of starch chains to digestive enzymes. In the case of HAS-PSs, the starch substrates were quickly hydrolyzed during the initial 40 min (Figure 8b). By extending the digestion to 120 min, the digested starch nearly reached 100% for all of the HAS-PSs, indicating a negligible content of RS. The increase of additional PS primarily enhanced the content of RDS in HAS-PSs (Table 1). Also, higher contents of RDS and SDS were observed for HAS-PSs than were observed in the control or corresponding HAS-SAs. Both micro- and macro-structures, in particular crystalline structures, affect the digestibility of starch. For example, it is well known that the crystalline structure is more resistant to amylolytic hydrolysis than the amorphous structure [4,25]. XRD analysis implied that the HAS-PSs showed higher V-type crystallinity but lower B-type crystallinity than the control or corresponding HAS-SAs. The results of rheology, FTIR, SAXS, and SEM suggest that the addition of PS restricted the reassociation of starch chains, which led to relatively loose structures of HAS-PSs. Thus, there were two possible pathways accounting for the improved digestibility of HAS-PSs. First, the V-type structure was more susceptible to amylolytic hydrolysis than the B-type structure, thus facilitating the hydrolysis of starch polymers. Second, the loosely structured HAS-PSs accelerated the diffusion of digestive enzymes to the starch substrates, which enhanced digestion. Assuming that the V-type crystalline structure dominated the digestion, then it was expected that the digestibility of starch would have a positive or negative correlation with V-type crystallinity. However, no significant difference in V-type crystallinity was observed in HAS-PS-1.0 (8.1%), HAS-PS-1.5 (12.1%), or HAS-PS-2.0 (16.1%). On the other hand, by increasing PS from 0.5 mM to 2.0 mM, the *Dm* of HAS-PSs gradually decreased from 2.56 to 2.15. Similar results were observed for the molecular order, as revealed by FTIR. Consequently, it was proposed that the molecular order of starch chains and the macro-structures of starch particles were more pertinent to the digestibility of starch-lipid complexes than the crystalline structures.

## 4. Conclusions

In conclusion, potassium stearate (PS) was easier to form V-type crystalline structures with amylose than stearic acid (SA) under the same conditions. The addition of PS decreased the reassociation of starch chains, leading to a low molecular order of starch chains and loose particle structures for HAS-PS. Relative to the V-type crystalline structure, the molecular order of starch chains (e.g., the alignment of double helices and B-type crystallinity) and the macro-structures of starch particles played a more important role in regulating the digestibility of starch-lipid complexes. The findings of the present study provide new insights into producing starch-lipid complexes and regulating the in vitro digestibility of starch-lipid complexes depending on their micro- and macro-structures.

## Figures and Tables

**Table 1:** Relative crystallinity, molecular order, mass fractal dimension, and in vitro digestibility of control, HAS-SAs, and HAS-PSs *.

Sample	XRD	FTIR		SAXS	In Vitro Digestibility		
	R_c_ (%) ^#^	1047/1016 Ratio	995/1016 Ratio	*D_m_* ^†^	RDS (%) ^‡^	SDS (%) ^‡^	RS (%) ^‡^
Control	20.0 ± 0.1 ^i^	0.38 ± 0.00 ^b,c^	1.07 ± 0.02 ^a^	2.96 ± ± 0.01 ^a^	36.7 ± 0.2 ^h^	33.0 ± 0.8 ^f^	30.3 ± 0.6 ^a^
HAS-SA-0.5 (3.6%)	22.7 ± 0.4 ^h^	0.41 ± 0.02 ^a^	1.09 ± 0.01 ^a^	2.96 ± 0.02 ^a^	44.7 ± 0.2 ^e^	39.2 ± 0.5 ^d^	16.1 ± 0.3 ^e^
HAS-SA-1.0 (7.1%)	25.6 ± 0.5 ^g^	0.40 ± 0.01 ^a^	1.08 ± 0.02 ^a^	2.95 ± 0.01 ^a^	37.8 ± 0.4 ^g^	35.9 ± 1.0 ^e^	26.3 ± 0.6 ^d^
HAS-SA-1.5 (10.7%)	28.9 ± 0.2 ^f^	0.41 ± 0.01 ^a^	1.07 ± 0.01 ^a^	2.95 ± 0.00 ^a^	38.8 ± 0.1 ^f^	33.1 ± 0.6 ^f^	28.1 ± 0.4 ^c^
HAS-SA-2.0 (14.2%)	33.2 ± 0.1 ^d^	0.41 ± 0.01 ^a^	1.08 ± 0.03 ^a^	2.96 ± 0.02 ^a^	39.3 ± 0.1 ^f^	31.5 ± 0.2 ^g^	29.2 ± 0.3 ^b^
HAS-PS-0.5 (4.0%)	30.1 ± 0.0 ^e^	0.40 ± 0.01 ^a, b^	1.01 ± 0.01 ^b^	2.56 ± 0.01 ^b^	46.5 ± 0.3 ^d^	53.4 ± 0.3 ^a^	0.2 ± 0.1 ^f^
HAS-PS-1.0 (8.1%)	34.3 ± 0.2 ^c^	0.36 ± 0.00 ^c^	0.84 ± 0.02 ^c^	2.42 ± 0.01 ^c^	47.6 ± 0.1 ^c^	52.5 ± 0.2 ^a, b^	0.1 ± 0.1 ^f^
HAS-PS-1.5 (12.1%)	35.5 ± 0.1 ^b^	0.37 ± 0.01 ^c^	0.85 ± 0.00 ^c^	2.30 ± 0.01 ^d^	48.5 ± 0.7 ^b^	51.2 ± 0.6 ^b^	0.1 ± 0.1 ^f^
HAS-PS-2.0 (16.1%)	36.1 ± 0.1 ^a^	0.36 ± 0.01 ^c^	0.84 ± 0.01 ^c^	2.15 ± 0.00 ^e^	50.1 ± 0.3 ^a^	49.7 ± 0.3 ^c^	0.1 ± 0.0 ^f^

* Means with different letters in the same column suggest significant differences (*p* < 0.05). ^#^ Rc: relative crystallinity. ^†^ *D_m_*: mass fractal dimension. ^‡^ RDS, SDS, and RS: rapidly digestible, slowly digestible, and resistant starches, respectively.

## Data Availability

The data presented in this study are available on request from the corresponding author due to privacy.

## Supplementary Materials

The following supporting information can be downloaded at https://www.mdpi.com/article/10.3390/foods13060859/s1. Figure S1: X-ray diffraction patterns of stearic acid (SA) and potassium stearate (PS); Figure S2: Enlarged FTIR spectra of HAS (control) and HAS-SAs (a), and HAS-PSs (b) at the range of 1200~800 cm^−1^. The HAS-SAs/PSs were prepared from dispersions containing 2.0% (*w*/*v*) high amylose starch (HAS) with stearic acid (SA) or potassium stearate (PS) at molar concentrations of 0.5~2.0 mM.

## References

[1] Oyeyinka S.A., Singh S., Amonsou E.O. (2021). A review on structural, digestibility and physicochemical properties of legume starch-lipid complexes. Food Chem..

[2] Ma R.R., Tian Y.Q., Zhang H.H., Cai C.X., Chen L., Jin Z.Y. (2019). Interactions between rice amylose and aroma compounds and their effect on rice fragrance release. Food Chem..

[3] Gao Q., Bie P.P., Tong X., Zhang B., Fu X., Huang Q. (2021). Complexation between High-Amylose Starch and Binary Aroma Compounds of Decanal and Thymol: Cooperativity or Competition?. J. Agric. Food Chem..

[4] Wang R., Zhang H., Chen Z.X., Zhong Q.X. (2020). Structural basis for the low digestibility of starches recrystallized from side chains of amylopectin modified by amylosucrase to different chain lengths. Carbohydr. Polym..

[5] D’Silva T.V., Taylor J.R.N., Emmambux M.N. (2011). Enhancement of the pasting properties of teff and maize starches through wet-heat processing with added stearic acid. J. Cereal Sci..

[6] Obiro W.C., Ray S.S., Emmambux M.N. (2012). V-amylose Structural Characteristics, Methods of Preparation, Significance, and Potential Applications. Food Rev. Int..

[7] Panyoo A.E., Emmambux M.N. (2017). Amylose-lipid complex production and potential health benefits: A mini-review. Starch-Stärke.

[8] Wang S.J., Chao C., Cai J.J., Niu B., Copeland L., Wang S. (2020). Starch-lipid and starch-lipid-protein complexes: A comprehensive review. Compr. Rev. Food. Sci. Food Saf..

[9] Zhang B., Huang Q., Luo F.-X., Fu X. (2012). Structural characterizations and digestibility of debranched high-amylose maize starch complexed with lauric acid. Food Hydrocoll..

[10] Englyst H.N., Kingman S.M., Cummings J.H. (1992). Classification and measurement of nutritionally important starch fractions. Eur. J. Clin. Nutr..

[11] DeMartino P., Cockburn D.W. (2020). Resistant starch: Impact on the gut microbiome and health. Curr. Opin. Biotech..

[12] Guo J.Y., Tan L.B., Kong L.Y. (2021). Impact of dietary intake of resistant starch on obesity and associated metabolic profiles in human: A systematic review of the literature. Crit. Rev. Food Sci..

[13] Gutiérrez T.J., Tovar J. (2021). Update of the concept of type 5 resistant starch (RS5): Self-assembled starch V-type complexes. Trends Food Sci. Techol..

[14] Hasjim J., Ai Y., Jane J.-L., Shi Y.C., Maningat C.C. (2013). Novel Applications of Amylose-Lipid Complex as Resistant Starch Type 5. Resistant Starch: Sources, Applications and Health Benefits.

[15] Marinopoulou A., Papastergiadis E., Raphaelides S.N., Kontominas M.G. (2016). Morphological characteristics, oxidative stability and enzymic hydrolysis of amylose-fatty acid complexes. Carbohydr. Polym..

[16] Shogren R.L., Fanta G.F., Felker F.C. (2006). X-ray diffraction study of crystal transformations in spherulitic amylose/lipid complexes from jet-cooked starch. Carbohydr. Polym..

[17] He W.-S., Wang Q., Zhao L., Li J., Li J., Wei N., Chen G. (2023). Nutritional composition, health-promoting effects, bioavailability, and encapsulation of tree peony seed oil: A review. Food Funct..

[18] Sun S., Jin Y., Hong Y., Gu Z., Cheng L., Li Z., Li C. (2021). Effects of fatty acids with various chain lengths and degrees of unsaturation on the structure, physicochemical properties and digestibility of maize starch-fatty acid complexes. Food Hydrocoll..

[19] Chao C., Yu J.L., Wang S., Copeland L., Wang S.J. (2018). Mechanisms Underlying the Formation of Complexes between Maize Starch and Lipids. J. Agric. Food Chem..

[20] Niu B., Chao C., Cai J.J., Yan Y.Z., Copeland L., Wang S., Wang S.J. (2019). The effect of NaCl on the formation of starch-lipid complexes. Food Chem..

[21] Wang R., Li Z.S., Zhang T.Q., Zhang H., Zhou X., Wang T., Feng W., Yu P.B. (2021). Impact of amylose content on the starch branch chain elongation catalyzed by amylosucrase from Neisseria polysaccharea. Food Hydrocoll..

[22] Zhang H., Wang R., Chen Z.X., Zhong Q.X. (2020). Amylopectin-Sodium Palmitate Complexes as Sustainable Nanohydrogels with Tunable Size and Fractal Dimensions. J. Agric. Food Chem..

[23] Nara S., Komiya T. (1983). Studies on the Relationship Between Water-satured State and Crystallinity by the Diffraction Method for Moistened Potato Starch. Starch-Stärke.

[24] Shang Y.Q., Chao C., Yu J.L., Copeland L., Wang S., Wang S.J. (2018). Starch Spherulites Prepared by a Combination of Enzymatic and Acid Hydrolysis of Normal Corn Starch. J. Agric. Food Chem..

[25] Zhang H., Wang R., Chen Z.X., Zhong Q.X. (2019). Enzymatically modified starch with low digestibility produced from amylopectin by sequential amylosucrase and pullulanase treatments. Food Hydrocoll..

[26] Zhou Y.G., Yang H.S. (2019). Effects of calcium ion on gel properties and gelation of tilapia (*Oreochromis niloticus*) protein isolates processed with pH shift method. Food Chem..

[27] Ding Y.Y., Cheng J.J., Lin Q.Y., Wang Q.Y., Wang J.R., Yu G.P. (2021). Effects of endogenous proteins and lipids on structural, thermal, rheological, and pasting properties and digestibility of adlay seed (*Coix lacryma-jobi* L.) starch. Food Hydrocoll..

[28] Karlberg M., Piculell L., Huang L. (2007). Solubility of amylose/ionic surfactant complexes in dilute aqueous solutions: Dependence on surfactant concentration. Carbohydr. Polym..

[29] Zobel H.F. (1988). Starch Crystal Transformations and Their Industrial Importance. Starch-Stärke.

[30] Li Q., Dong Y.Y., Gao Y., Du S.K., Li W.H., Yu X.Z. (2021). Functional Properties and Structural Characteristics of Starch-Fatty Acid Complexes Prepared at High Temperature. J. Agric. Food Chem..

[31] Fanta G.F., Shogren R.L., Salch J.H. (1999). Steam jet cooking of high-amylose starch fatty acid mixtures. An investigation of complex formation. Carbohydr. Polym..

[32] Ji N., Qin Y., Li M., Xiong L., Qiu L.Z., Bian X.L., Sun Q.J. (2018). Fabrication and Characterization of Starch Nanohydrogels via Reverse Emulsification and Internal Gelation. J. Agric. Food Chem..

[33] Cervantes-Ramirez J.E., Cabrera-Ramirez A.H., Morales-Sanchez E., Rodriguez-Garcia M.E., Reyes-Vega M.D., Ramirez-Jimenez A.K., Contreras-Jimenez B.L., Gaytan-Martinez M. (2020). Amylose-lipid complex formation from extruded maize starch mixed with fatty acids. Carbohydr. Polym..

[34] Lopez-Rubio A., Flanagan B.M., Shrestha A.K., Gidley M.J., Gilbert E.P. (2008). Molecular rearrangement of starch during in vitro digestion: Toward a better understanding of enzyme resistant starch formation in processed starches. Biomacromolecules.

[35] Zhang H., Qian S., Rao Z.M., Chen Z.X., Zhong Q.X., Wang R. (2021). Supermolecular structures of recrystallized starches with amylopectin side chains modified by amylosucrase to different chain lengths. Food Hydrocoll..

[36] Yang S., Zhu M.P., Wang N., Cui X.T., Xu Q., Saleh A.S.M., Duan Y.M., Xiao Z.G. (2018). Influence of Oil Type on Characteristics of -Sitosterol and Stearic Acid Based Oleogel. Food Biophys..

[37] Zabar S., Lesmes U., Katz I., Shimoni E., Bianco-Peled H. (2009). Studying different dimensions of amylose-long chain fatty acid complexes: Molecular, nano and micro level characteristics. Food Hydrocoll..

[38] Schaefer D.W., Keefer K.D. (1984). Fractal Geometry of Silica Condensation Polymers. Phys. Rev. Lett..

[39] Suzuki T., Chiba A., Yano T. (1997). Interpretation of small angle X-ray scattering from starch on the basis of fractals. Carbohydr. Polym..

[40] Zhang H., Zhou X., He J., Wang T., Luo X.H., Wang L., Wang R., Chen Z.X. (2017). Impact of amylosucrase modification on the structural and physicochemical properties of native and acid-thinned waxy corn starch. Food Chem..

