# Supplementary material for: Structural Features, Physicochemical Properties, and In Vitro Digestibility of the Starch-Lipid Complexes Formed between High Amylose Starch and Stearic Acid or Potassium Stearate
Source: Foods. 2024 Mar 12;13(6):859. doi: 10.3390/foods13060859 (PMC10969461; doi:10.3390/foods13060859)

Supplementary material

Figure S1. X-ray diffraction patterns of stearic acid (SA) and potassium stearate (PS).

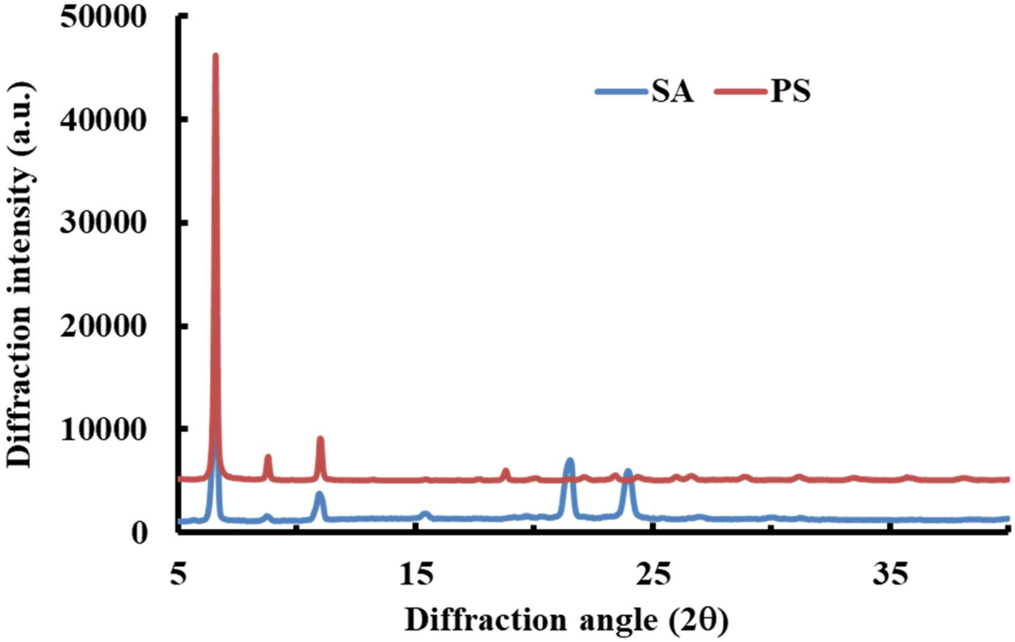

Figure S2. Enlarged FTIR spectra of HAS (control) and HAS-SAs (a), and HAS-PSs (b) at the range of 1200~800 cm^{-1}. The HAS-SAs/PSs were prepared from the dispersions containing 2.0 % (w/v) high amylose starch (HAS) with stearic acid (SA) or potassium stearate (PS) at molar concentrations of 0.5~2.0 mM.

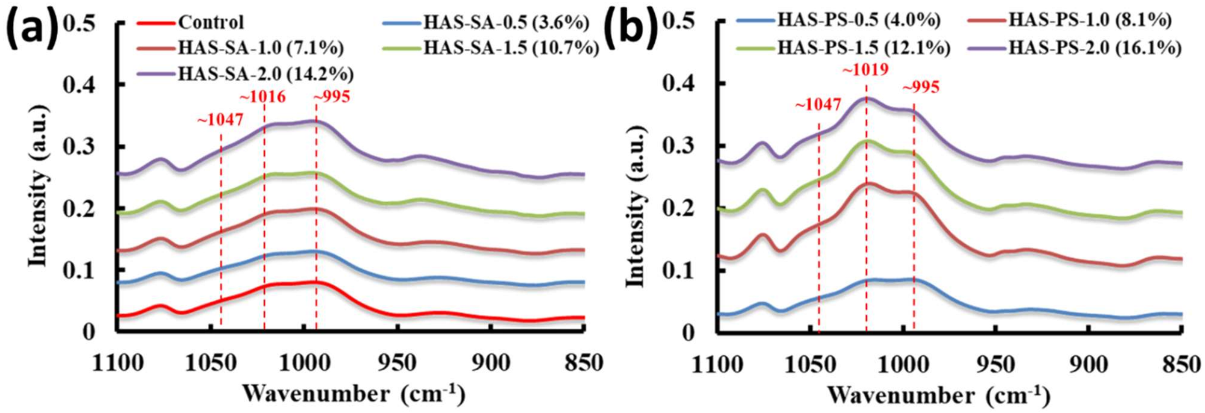

Supplement: Supplementary file 1 [file foods-13-00859-s001.zip › foods-2887507-supplementary.pdf]
